# Fisetin Attenuates Uterine Leiomyoma in Rats: Potential Crosstalk Between HMGB1, SphK1/S1P, and TGF‐β Signaling

**DOI:** 10.1002/jbt.71105

**Published:** 2026-08-31

**Authors:** Nashwa Maghraby, Esraa Khalaf, Marwa T. Hussien, Ahmed M. A. Sobh, Nagla T. Elmelegy

**Affiliations:** ^1^ Department of Medical Biochemistry and Molecular Biology, Faculty of Medicine Assiut University Assiut Egypt; ^2^ Department of Basic Medical Sciences Badr University of Assiut (BUA) New Nasser City Egypt; ^3^ Department of Oncologic Pathology, South Egypt Cancer Institute Assiut University Assiut Egypt; ^4^ Department of Obstetrics and Gynecology Assiut University Assiut Egypt

**Keywords:** fisetin, high mobility group box 1, sphingosine 1‐phosphate, sphingosine kinase 1, TGF‐β, uterine leiomyomas

## Abstract

Uterine leiomyomas (UL) represent one of the most prevalent gynecological benign tumors with a considerable global medical and economic burden. Effective therapeutic strategies that preserve the uterus and maintain fertility are still lacking. Fisetin (FIS), a naturally occurring dietary flavonoid, exhibits antitumor, anti‐inflammatory, and antifibrotic properties, yet its therapeutic potential in UL remains incompletely defined. This study investigated the therapeutic effects of fisetin on UL and the possible underlying mechanisms. UL was experimentally induced in adult female rats by monosodium glutamate (MSG), and animals were assigned to Control, FIS, UL, and UL + FIS groups. Blood and tissue samples were subjected to hormonal, biochemical, histopathological, and immunohistochemical assessments. Serum estradiol, progesterone, and S1P levels were measured using ELISA, while qPCR quantified the expression of key signaling molecules, including SphK1, TGF‐β1, HMGB1, TNF‐α, and IL‐1β. Immunohistochemistry assessed TGF‐β1 and α‐SMA expressions, and histopathology (H&E and Masson's Trichrome stains) evaluated tissue architecture and fibrosis. The results showed that fisetin administration significantly reduced the elevated sex hormones, inhibited S1P signaling, and suppressed proinflammatory mediators (HMGB1, TNF‐α, IL‐1β) and profibrotic mediators (TGF‐β1, α‐SMA). Histopathological evaluation confirmed decreased fibrotic remodeling and improved tissue architecture. Overall, these results suggest that fisetin's effect may involve coordinated modulation of the S1P/SphK1, TGF‐β, and HMGB1 axes, highlighting its potential as a novel uterus‐preserving therapeutic agent for UL.

## Graphical abstract

Fisetin attenuates monosodium glutamate‐induced uterine leiomyoma by reducing sex hormones and suppressing S1P/SphK1 signaling, HMGB1‐mediated inflammation, and fibrotic markers. These coordinated effects alleviate fibrosis and restore uterine tissue architecture, highlighting fisetin as a promising natural therapeutic candidate.

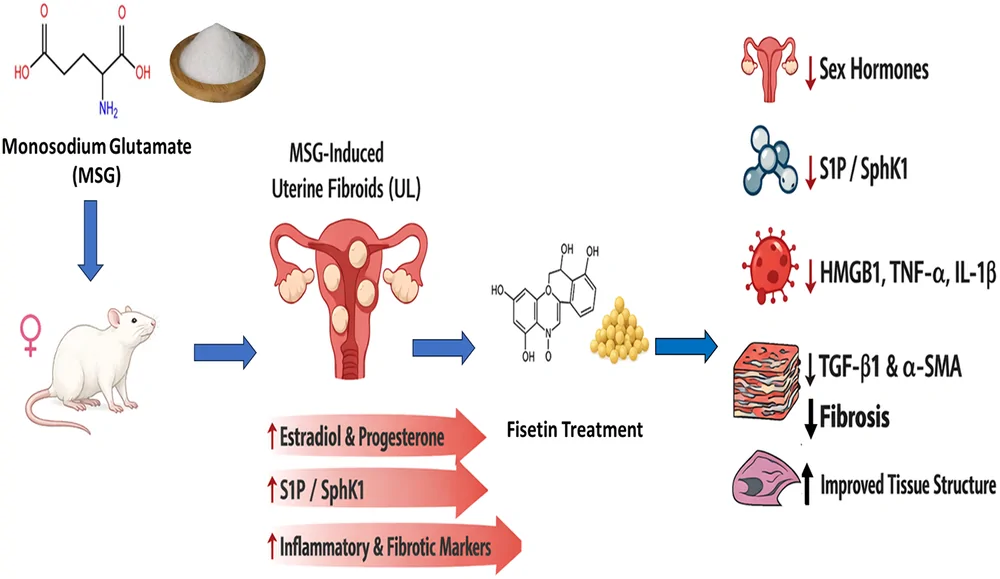

**Abbreviations**
α‐SMA: alpha‐smooth muscle actin
(COCs): combined oral contraceptives
ELISA: enzyme‐Linked immunosorbent assay
FIS: fisetin
(GnRH): gonadotropin‐releasing hormone agonists
HMGB1: high‐mobility group box 1
H&E: hematoxylin and eosin
IL‐1β: interleukin1beta
MSG: monosodium glutamate
qPCR: quantitative polymerase chain reaction
S1P: sphingosine 1‐phosphate
TGF‐β1: transforming growth factor β1
TNF‐α: Tumor necrosis factor alpha
UL: uterine leiomyoma

## 1 Introduction

Uterine leiomyoma (UL), commonly known as a uterine fibroid, represents the most prevalent benign pelvic tumor originating from the smooth muscle layer of the uterine myometrium, predominantly observed in women within their fertile years [1]. Approximately 70%–80% of women will experience UL before their fifth decade of life [2]. UL burden is increasing globally, affecting up to 50% of cases, causing debilitating symptoms like infertility, bleeding, anemia, pelvic pressure, pain, constipation, miscarriage, and preterm birth, reflecting the profound effect of UL on their quality of life [3].

Although the exact molecular and cellular mechanisms involved in the development of UL are not yet fully understood, accumulating evidence implicates that genetic factors, growth factors, inflammation, steroid hormones, and excessive accumulation of extracellular matrix (ECM) are key contributors to UL growth [4]. It is widely recognized that estrogen and progesterone, the gonadal sex steroid hormones, are key regulators of UL formation, with their effects mainly mediated by locally produced growth factors, particularly transforming growth factor β (TGFβ) [5]. TGF‐β is a pivotal cytokine/growth factor that stimulates fibroblasts and hastens ECM production in injured tissues. It not only activates fibroblasts and stimulates myofibroblast differentiation, but also promotes fibroblast proliferation [6]. Moreover, it regulates a wide range of processes, such as apoptosis, angiogenesis, and tumor development, highlighting its pivotal role in the pathophysiology of UL [7].

High‐mobility group box 1 (HMGB1) is a member of a class of non‐histone DNA‐binding proteins that is mainly localized in the nucleus, and regulates DNA events such as DNA repair and genome stability [8, 9]. When released into the extracellular space, it binds to the receptor for advanced glycation end products (RAGE) located on the cell membrane, triggering a cascade of signals that regulate key processes such as cell proliferation, autophagy, inflammation, and immunity [10]. HMGB1 is involved in key aspects of cancer biology, such as uncontrolled growth, angiogenesis, inflammation, resistance to apoptosis, self‐sufficiency in growth signals, as well as invasion and metastasis potential [11]. Notably, HMGB levels are elevated in the UL tissues compared to paraneoplastic tissues, suggesting that it promotes UL cell proliferation [12].

Sphingolipid signaling has recently emerged as an important mediator of the rapid signaling effects of estrogen [13]. Sphingosine 1‐phosphate (S1P), a serum‐borne bioactive lipid mediator, is generated by the enzymes sphingosine kinase 1 and 2, SphK1 and SphK2 [14]. After its production, S1P can play its role either as an intracellular messenger or in paracrine/autocrine pathways by binding to its membrane receptors 1–5 (S1PR1–5) [15]. SphK1/S1P is upregulated in cancer cells and linked to tumor progression, including UL [16]. Notably, SphK1 inhibition was found to suppress both HMGB1 expression and intracellular translocation, highlighting their functional link [17].

Current treatments of UL include invasive and non‐invasive methods like myomectomy, hysterectomy, hormone replacement therapy, and uterine artery embolization [18]. However, these methods have risks, side effects, high recurrence rates, and high healthcare costs [2], necessitating the development of novel agents. Fisetin (3,7,3,4‐tetrahydroxyflavone) is a naturally occurring non‐toxic flavonoid found in various fruits and vegetables such as strawberries, apples, and persimmons [19]. It possesses powerful antioxidant, anti‐inflammatory, and antifibrotic properties, and recent studies indicate that it also has anti‐proliferative and apoptotic effects in various cancer cell lines [20, 21], supporting its potential as a novel therapeutic agent for UL.

Considering the antioxidant, anti‐inflammatory, anti‐proliferative, and anti‐fibrotic properties of fisetin, we hypothesized that it could attenuate UL growth and ECM accumulation. Accordingly, the current study aimed to evaluate the therapeutic potential of fisetin in an MSG‐induced rat model of UL and to investigate the associated molecular alterations in key signaling pathways implicated in UL pathogenesis, including HMGB1, S1P/SphK1, and TGF‐β signaling.

## 2 Materials and Methods

### 2.1 Experimental Animals

Forty healthy, nonpregnant adult female Wistar rats weighing 160 ± 10 grams (10–12 weeks) were used in this experiment. They were obtained from the Animal House of the Faculty of Medicine, Assiut University. They were housed in metal cages according to the study's group classification and allowed to acclimate for 2 weeks before the experiment. The animals were maintained in good condition at 22 ± 2°C and 50 ± 5% humidity, on a natural light‐dark cycle, with free access to food and water. The Ethics Committee of the Faculty of Medicine, Assiut University, Assiut, Egypt, approved all experimental procedures (Approval No. 04‐2023/200221).

### 2.2 Chemicals

**Monosodium glutamate (MSG)** was obtained from CHEMLAB Chemical Technology as a commercial formulation C5H8NO4Na in the form of white crystalline granules. Every week, a fresh solution was prepared by dissolving 3.7 g of MSG salt in 50 ml of D.W. Fisetin was obtained from Sigma‐Aldrich, St. Louis, USA, in the form of fine yellow powder. It was freshly prepared by dissolving in 0.5% sodium carboxymethyl cellulose (CMC‐Na).

### 2.3 Animal Grouping

After the period of acclimatization, the rats were divided into 4 equal groups of 10 animals each:

**Group I (Normal Control Group):** Rats subjected to neither leiomyoma induction nor fisetin treatment.

**Group II (FIS Group):** Rats were orally administered fisetin at a dose of 50 mg/kg/day with a gavage volume of 10 mL/kg for 1 month from day 30 to day 60.

**Group III (UL Group):** Rats were subjected to leiomyoma induction using a single daily dose of 800 mg/kg MSG for 30 days [22], Then stopped, with normal rat chow, continued till day 60.

**Group IV (UL + FIS):** Rats were subjected to leiomyoma induction, then orally treated with fisetin at a dose of 50 mg/kg/day with a gavage volume of 10 mL/kg for another month [23].

Rats in the control and model groups were administered 0.5% CMC‐Na solution with a gavage volume of 10 mL/kg for the same duration as the FIS and UL + FIS groups.

### 2.4 Establishment of UL Model

UL was induced with a single daily dose of 800 mg MSG/kg over 30 days using an oral gavage tube, following the protocol described by Asante et al [22]. All treatments were conducted between 9:00 and 10:00 a.m. UL development was confirmed by laparotomy, supported by a histopathological evaluation and hormonal measurements.

Following the induction period, samples of blood and uterine tissue were collected from animals in both control and UL groups. Blood samples were analyzed to assess estradiol and progesterone levels. Animals in the UL group showed noticeably elevated hormone levels compared to the control animals. This rise in steroid hormones supports earlier studies showing that increased estradiol and progesterone are characteristic markers of UL [2, 24].

Regarding the uterine tissue samples, they were processed using standard histological methods, including routine Hematoxylin and Eosin (H&E) staining. This was done to confirm the successful induction of tumors before beginning the treatment. The confirmation of UL induction was based on the detection of densely packed, spindle cell‐shaped muscles and fibrous tissue with increased thickness in comparison to the control group.

### 2.5 Sample Collection

At the end of the experiment, before sacrifice, animals were precisely weighed and anesthetized. Blood samples were collected from the retro‐orbital sinus and centrifuged at 3000 rpm for 15 min. The serum was promptly separated and stored at −20°C until estradiol, progesterone, and S1P levels were measured. Subsequently, the anesthetized animals were euthanized by decapitation. Following cleansing of the abdominal skin and peritoneum with 70% ethanol, the uterus was carefully dissected out using scissors and forceps, quickly rinsed with ice‐cold phosphate‐buffered saline, and weighed.

The most intumescent part of the uterus was obtained, and the uterine tissues of each rat were divided into two parts: One part was snap frozen in liquid nitrogen and stored at −80°C for later biochemical measurements. The Other part was preserved using 10% neutral buffered formalin placed in pre‐labeled containers for histopathological and immunohistochemical analysis.

### 2.6 Uterine Coefficient Calculation and Gross Organ Morphology

At the end of the experiment, the body weight (BW) of each animal was recorded. Following the animal scarification and tissue collection, the uterine weight of each animal was measured. These measurements were used to determine the uterine coefficient, calculated as uterine weight divided by body weight × 100. Additionally, photographs of uteri were captured to illustrate the gross morphological differences among the experimental groups.

### 2.7 Quantitative Real‐Time Polymerase Chain Reaction (qRT‐PCR)

Frozen uterine tissue samples were first homogenized, and then total RNA was extracted using the RNeasy Mini Kit (catalog no. 74104, Qiagen, Germany). Subsequently, 500 ng of extracted RNA were reverse transcribed into cDNA using a High‐Capacity cDNA Reverse Transcription Kit with RNase Inhibitor (Cat. No. 4374966, Thermo Fisher Scientific, USA). The expression levels of selected genes (*Sphk1, HMGB1, TNF‐α, TGFβ1 and IL‐1β*) were determined by quantitative reverse transcriptase polymerase chain reaction on a on a 7500 Fast Real‐Time PCR System (Applied Biosystems, Foster City, CA, USA) with the use of using Maxima SYBR Green qPCR Master Mix (2x) (Catalog no. #K0251, Thermo‐Fisher Scientific, USA) and specific primers (Table 1). The β‐actin served as an internal control. The 2^−ΔΔCt^ method was used for evaluating the relative expression [25].

**Table 1:** Primers used for qRT‐PCR reaction.

Gene	5′–3′ primer sequence	Accession number
β‐actin	Forward: CACTATCGGCAATGAGCGGTTCC	NM_031144.3
	Reverse: CAGCACTGTGTTGGCATAGAGGTC	
SphK1	Forward: CGCCTGGGCAACACCGATAA	NM_001270809.1
	Reverse: GGCTACATAGGGGTTTCTGG	
HMGB1	Forward: AGATGACAAGCAGCCCTAT	XM_039100280.2
	Reverse: CTTTTCAGCCTTGACCAC	
TGF‐β1	Forward: GACCGCAACAACGCAATCTATGAC	NM_021578.2
	Reverse: CTGGCACTGCTTCCCGAATGTC	
TNF‐α	Forward: ACCACGCTCTTCTGTCTACTG	NM_013693.3
	Reverse: CTTGGTGGTTTGCTACGAC	
IL‐1β	Forward: GACTTCACCATGGAACCCGT	NM_031512.2
	Reverse: GGAGACTGCCCATTCTCGAC	

### 2.8 Enzyme‐Linked Immunosorbent Assays (ELISA)

After centrifugation, serum samples were thawed at 4°C, diluted, and analyzed for estradiol (E2), progesterone (P), and S1P levels using commercial ELISA kits. Serum E2‐ and p levels were quantified using ELISA kits purchased from CUSABIO, USA (Cat. No. CSB‐E05110r and CSB‐E07282r for E2 and P, respectively), while serum levels of S1P were quantified using ELISA kits purchased from Cloud‐Clone Corp (Cat. No. CEG031Ge). ELISA procedures were conducted according to the instructions provided in the commercial kits.

### 2.9 Histopathological and Immunohistochemical Examination

Uterine tissues from all groups were fixed in 10% neutral‐buffered formalin. Once fixed, specimens were dissected, processed, and embedded in paraffin to prepare formalin‐fixed, paraffin‐embedded (FFPE) tissue blocks. Sections of 4 µm‐thickness were serially cut from each block and stained with hematoxylin and eosin (H&E) stain and with Masson's trichrome stain. The stained slides were examined under a light microscope, and the photomicrographs were subsequently evaluated using the Image‐J2X software.

For immunohistochemistry analysis, formalin‐fixed, paraffin‐embedded tissue slices were stained for TGF‐β1 and α‐SMA antibodies supplied by Abclonal Inc., China (Cat. No: A16640 and A17910, for TGF‐β1 and α‐SMA, respectively) according to the manufacturer's instructions. Immunohistochemical staining was performed using the streptavidin‐biotin–peroxidase complex method [26].

### 2.10 Statistical Analysis

Statistical analysis was performed using GraphPad Prism software version 8.0.1. The normal distribution of the data within each group was assessed using the Shapiro‐Wilk test. A one‐way analysis of variance (ANOVA) was utilized to analyze the differences between groups, followed by post hoc Tukey's Honestly Significant Difference (HSD) test. After the induction period, a *t*‐test was used to compare estradiol and progesterone levels between the model group and the control group. All data are presented as mean ± standard deviation (SD). A *p*‐value less than 0.05 was considered statistically significant.

## 3 Results

### 3.1 Body Weights and the Uterine Coefficients

As illustrated in Table 2, the body weight was similar across groups, with only a slight increase in the model group. Regarding the uterine weight uterine coefficient, there was a significant increase in the uterine coefficient between the control and UL groups (*p* < 0.0001). On the other hand, the uterine coefficient was decreased significantly in the UL + FIS group (*p* < 0.0001) compared to the UL group.

**Table 2:** Body weights, uterine weights, and the uterine coefficients.

Variables	Control (*N* = 10)	FIS (*N* = 10)	UL (N = 10)	UL + FIS (N = 10)
Uterine weight (g)	0.38 ± 0.03	0.39 ± 0.04	0.77 ± 0.1^a^, ***	0.47 ± 0.04^a^, ^b^, ^c^, *, **, ***
Final body weight (g)	208.1 ± 14.65	210.5 ± 15.23	219.5 ± 20.81	205.2 ± 5.10
Uterine weight/body weight (%)	0.18 ± 0.01	0.19 ± 0.02	0.35 ± 0.06^a^, ***	0.23 ± 0.02^a^, ^b^, ^c^, *, **, ***

*Note:* Data are expressed as mean ± SD (*n* = 10 in each group) and analyzed by One‐way analysis of variance (ANOVA) followed by the Turkey test.

Abbreviations: FIS: fisetin group; UL: uterine leiomyoma group; UL + FIS: uterine leiomyoma + fisetin group.

^a^ Significantly different from the value in the control group.

^b^ Significantly different from the value in the FIS group.

^c^ Significantly different from the value in the UL group.

* *p* ≤ 0.05

** *p* ≤ 0.01, and

*** *p* ≤ 0.001.

### 3.2 Morphological Changes of the Uterus

**Figure 1:**
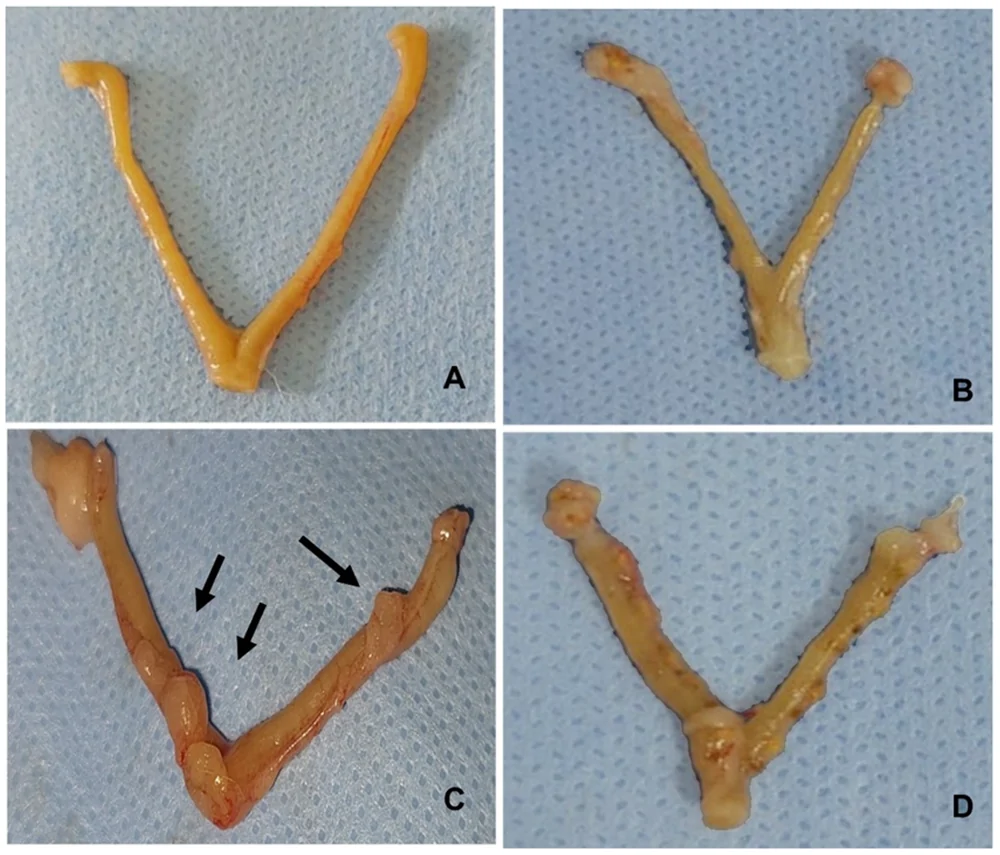
Gross morphological images showing the uterus of groups (A) Control, (B) FIS, (C) UL, (D) UL + FIS. black arrows: swellings and nodules suspected intramural and pedunculated fibroids. FIS, fisetin; UL, uterine leiomyoma; UL + FIS, uterine leiomyoma + fisetin.

As shown in (Figure 1), rats in the control and FIS groups showed that the uterine horns were symmetrical, Y‐shaped, uniform in appearance, without any cystic changes or swellings. In contrast, the model group exhibited shorter and asymmetrical uterine horns characterized by firm consistency and nodular swellings. Conversely, treatment of the UL model rats with fisetin reduced the size of the uterine horn and noticeably improved most of the abnormal features, bringing them closer to normal.

### 3.3 Serum Levels of Estradiol and Progesterone

**Figure 2:**
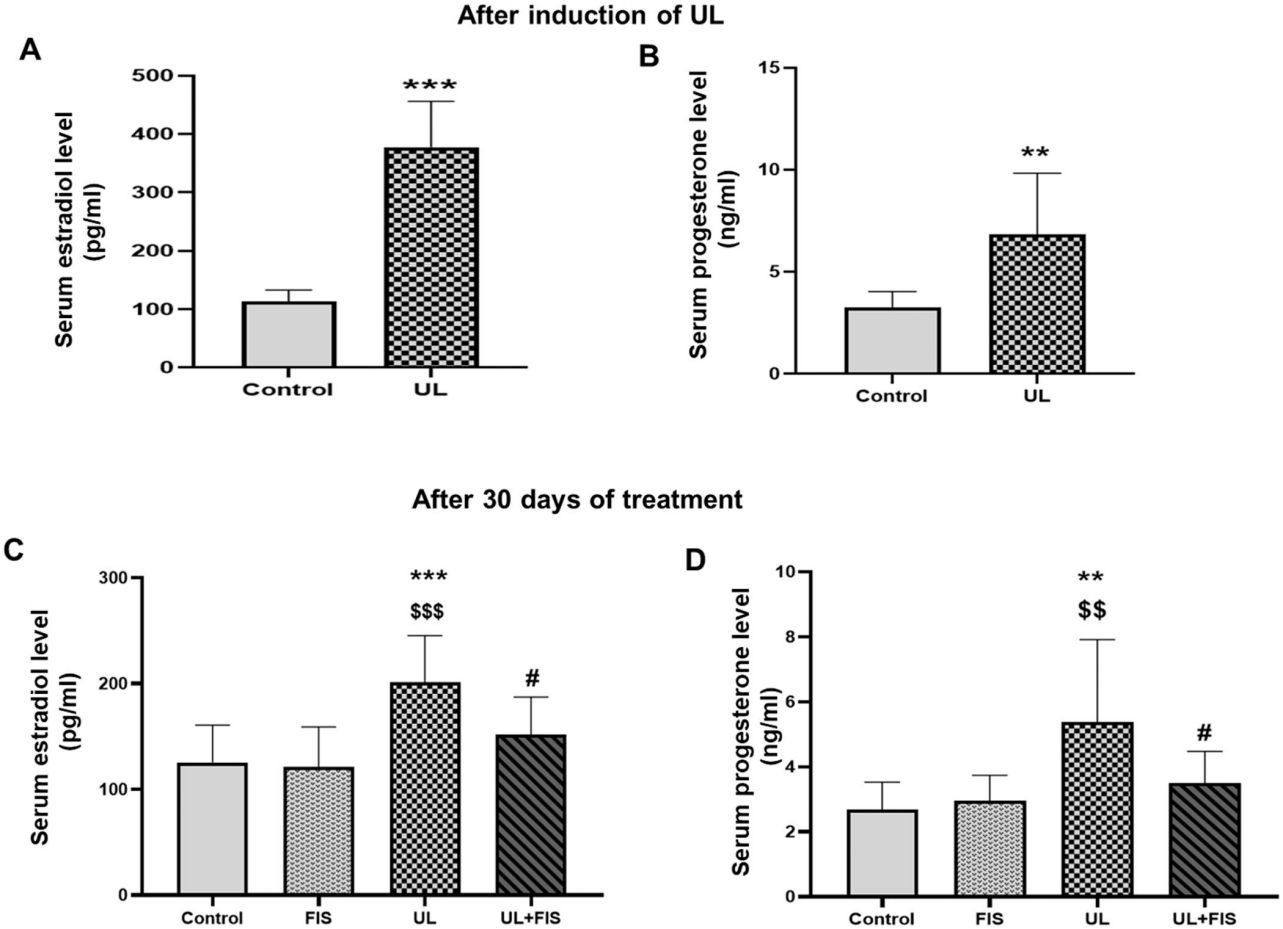
Serum levels of estradiol and progesterone among different groups. (A, B): After induction of UL (C, D): At the end of the experiment. *n* = 10 in each group. Data are expressed as mean ± SD. **p ≤ 0.01, ***p ≤ 0.001 versus control group; ^$$^ *p* ≤ 0.01, ^$$$^ *p* ≤ 0.001 versus FIS‐treated group; ^#^ *p* ≤ 0.05, versus UL group. FIS, fisetin; UL, uterine leiomyoma; UL + FIS, uterine leiomyoma + fisetin.

Following the induction phase, the UL group revealed significantly higher estradiol and progesterone concentrations (*p* < 0.0001 and 0.0019, respectively) compared to controls. This elevation persisted until the end of the experiment, with model animals maintaining higher estradiol and progesterone levels than controls (*p* = 0.0005 and 0.0013, respectively). Notably, after 30 days of fisetin treatment, the previously increased serum estradiol and progesterone levels significantly declined compared to the UL group (*p* = 0.0307 and 0.036, respectively) (Figure 2).

### 3.4 Effect of Fisetin on SphK1/S1P Axis

To explore the effect of fisetin on the SphK1/S1P axis in uterine leiomyomas, the serum level of S1P, as well as the expression level of the enzyme that produces SphK1, was assessed. According to the real‐time PCR results, SphK1 expression was significantly higher (*p* < 0.0001) in the uterine tissues of the UL group compared to those of the control group. On the other hand, 30 days of fisetin treatment significantly decreased SphK1 expression in the uterine tissues of the UL + FIS group (*p* = 0.0015) as compared to the model group.

**Figure 3:**
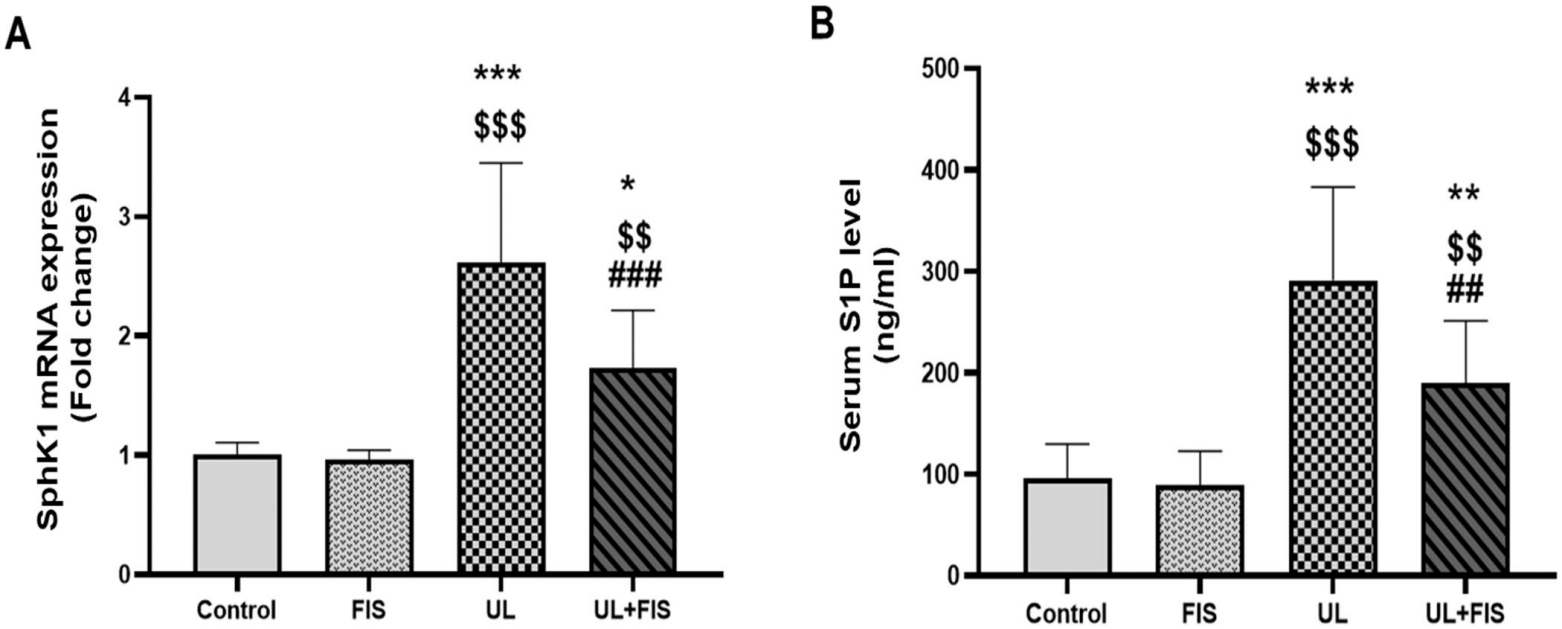
Effect of fisetin on SphK1/S1P axis in uterine leiomyomas. (A) SphK1 relative mRNA expression (B) serum level of S1P. *n* = 10 in each group. β‐actin was utilized to normalize expression data. Data are expressed as mean ± SD. *p ≤ 0.05, **p ≤ 0.01,***p ≤ 0.001 versus control group; ^$$^ *p* ≤ 0.01, ^$$$^ *p* ≤ 0.001 versus FIS‐treated group; ^##^ *p* ≤ 0.01, ^###^ *p* ≤ 0.001 versus UL group. FIS, fisetin; SphK1, sphingosine kinase 1; S1P, sphingosine 1 phosphate; UL, uterine leiomyoma; UL + FIS, uterine leiomyoma + fisetin.

Consistently, the plasma level of S1P was significantly (*p* < 0.0001) higher in the UL group compared to those in the control group. Instead, its level significantly decreased in the UL + FIS group as compared to its level in the UL group (*p* = 0.0032) (Figure 3).

### 3.5 Effect of Fisetin on HMGB1, and Inflammatory Cytokines (IL‐1β and TNF‐α)

**Figure 4:**
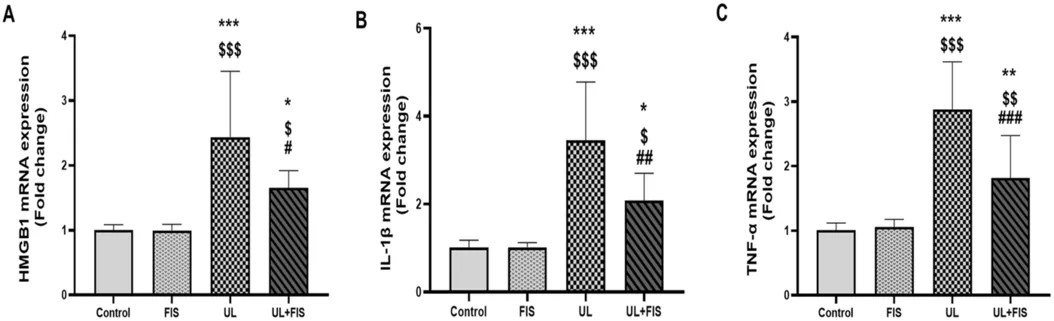
Effect of fisetin on the relative mRNA expression of (A) HMGB1, (B) IL‐1β, (C) TNF‐α in UL. *n* = 10 in each group. β‐actin was utilized to normalize expression data. Data are expressed as mean ± SD. *p ≤ 0.05, **p ≤ 0.01, ***p ≤ 0.001 versus control group; ^$^ *p* ≤ 0.05, ^$$^ *p* ≤ 0.01, ^$$$^ *p* ≤ 0.001 versus FIS‐treated group; ^#^ *p* ≤ 0.05, ^##^ *p* ≤ 0.01, ^###^ *p* ≤ 0.001 versus UL group. FIS, fisetin; HMGB1, High‐mobility group box 1; IL‐1β, interleukin1β; TNF‐α, tumor necrosis factor α; UL, uterine leiomyoma; UL + FIS, uterine leiomyoma+ fisetin.

The results showed that the expression levels of HMGB1, IL‐1β, and TNF‐α were significantly increased (*p* < 0.0001) in the uterine tissues of the model group compared to the normal uterine tissues of the control group. Moreover, administration of 50 mg/kg of fisetin resulted in significant decreases in the expression levels of HMGB1, IL‐β, and TNF‐α (*p* = 0.0112, 0.0011, and 0.0002, respectively) compared to the induction group (Figure 4).

### 3.6 Effect of Fisetin on Fibrosis Biomarkers (TGF‐β1 and α‐SMA)

**Figure 5:**
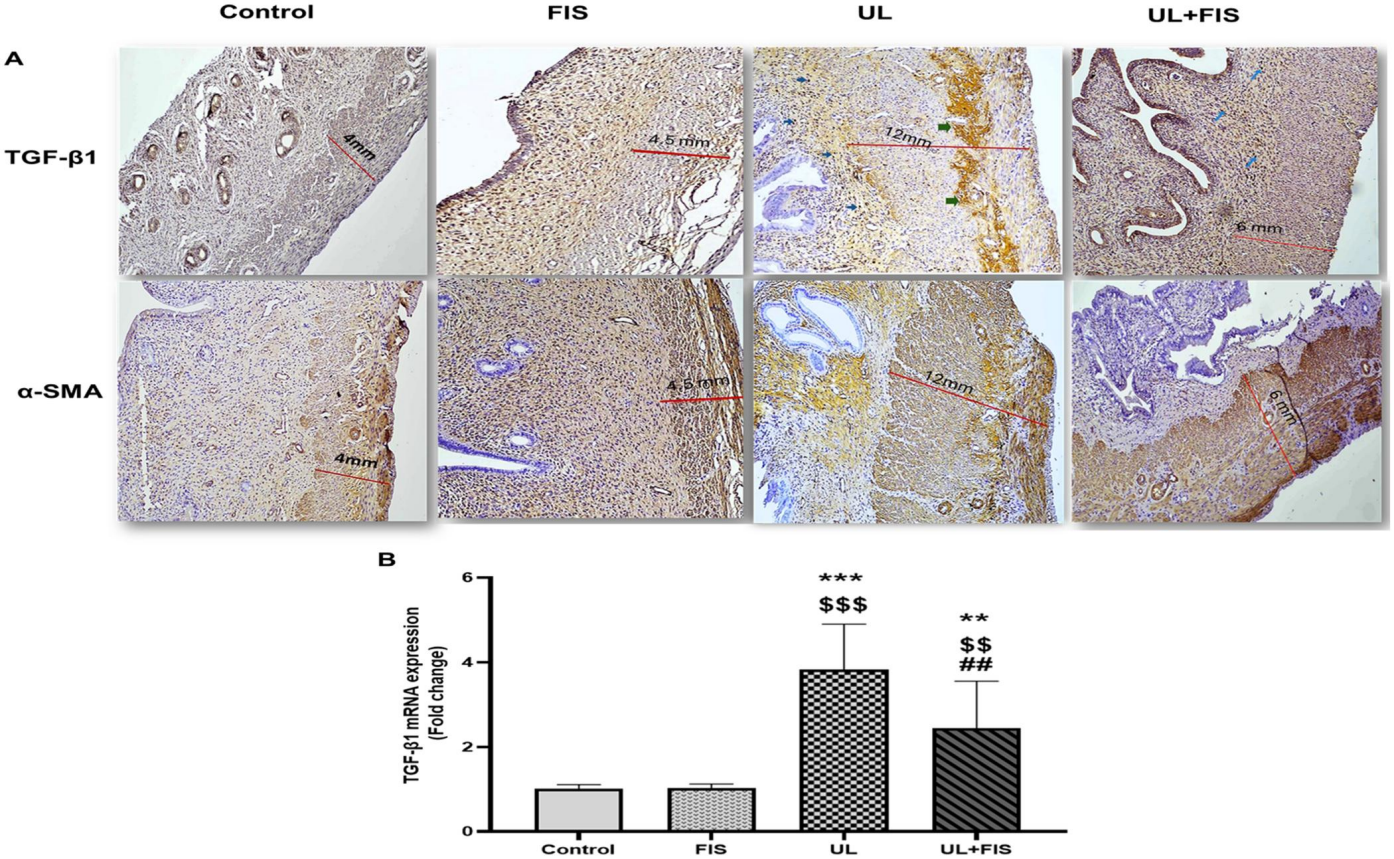
Effect of fisetin on fibrosis biomarkers (TGF‐β1 and α‐SMA). (A) Immunostaining of TGF‐β1 and α‐SMA in the uterine tissues of different animal groups. In the control and FIS groups, TGF‐β1 expression was minimal in stromal cells and mononuclear immune cells. In the induction group, TGF‐β1 expression was prominent in stromal cells between muscle (green arrow) and within immune cells in the endometrial tissue (blue arrow). The UL + FIS group exhibited a significant decrease in TGF‐β1 expression in stromal and immune cells (blue arrow). Regarding α‐SMA, its expression in smooth muscle cells highlights myometrial thickness of approximately 4 mm and 4.5 mm in the control and FIS groups, respectively. In the induction group, α‐SMA expression indicates a notable increase in myometrial thickness, reaching 12 mm. In the UL + FIS group, α‐SMA expression shows a substantial reduction in myometrial thickness compared to the induction group, decreasing to about 6 mm (×40). (B) Relative mRNA expression of TGF‐β1. *n* = 10in each group. β‐actin was used to normalize expression data. Data are presented as means ± S.D. **p ≤ 0.01, ***p ≤ 0.001 versus control group; ^$$^ *p* ≤ 0.01, ^$$$^ *p* ≤ 0.001 versus FIS‐treated group; ^##^ *p* ≤ 0.01 versus UL group. FIS, fisetin; TGF‐β1, transforming growth factor β1; α‐SMA, α‐ α‐smooth muscle actin; UL, uterine leiomyoma; UL + FIS, uterine leiomyoma + fisetin.

As shown in Figure 5, the results revealed a significantly higher mRNA expression of TGF‐β1 in the uterine tissues of the model group compared to the control group (*p* < 0.0001). However, fisetin treatment significantly reduced (*p* = 0.0017) the expression of this marker relative to the induction group.

These results were further confirmed by immunohistochemical analysis of TGF‐β1 and α‐SMA expressions. The Analysis showed strong immunoreactivity for TGF‐β1 and α‐SMA in the induction group as compared to the control group. Conversely, fisetin treatment reversed the expression of these markers to varying degrees as compared with the model group.

### 3.7 Histopathological Results

**Figure 6:**
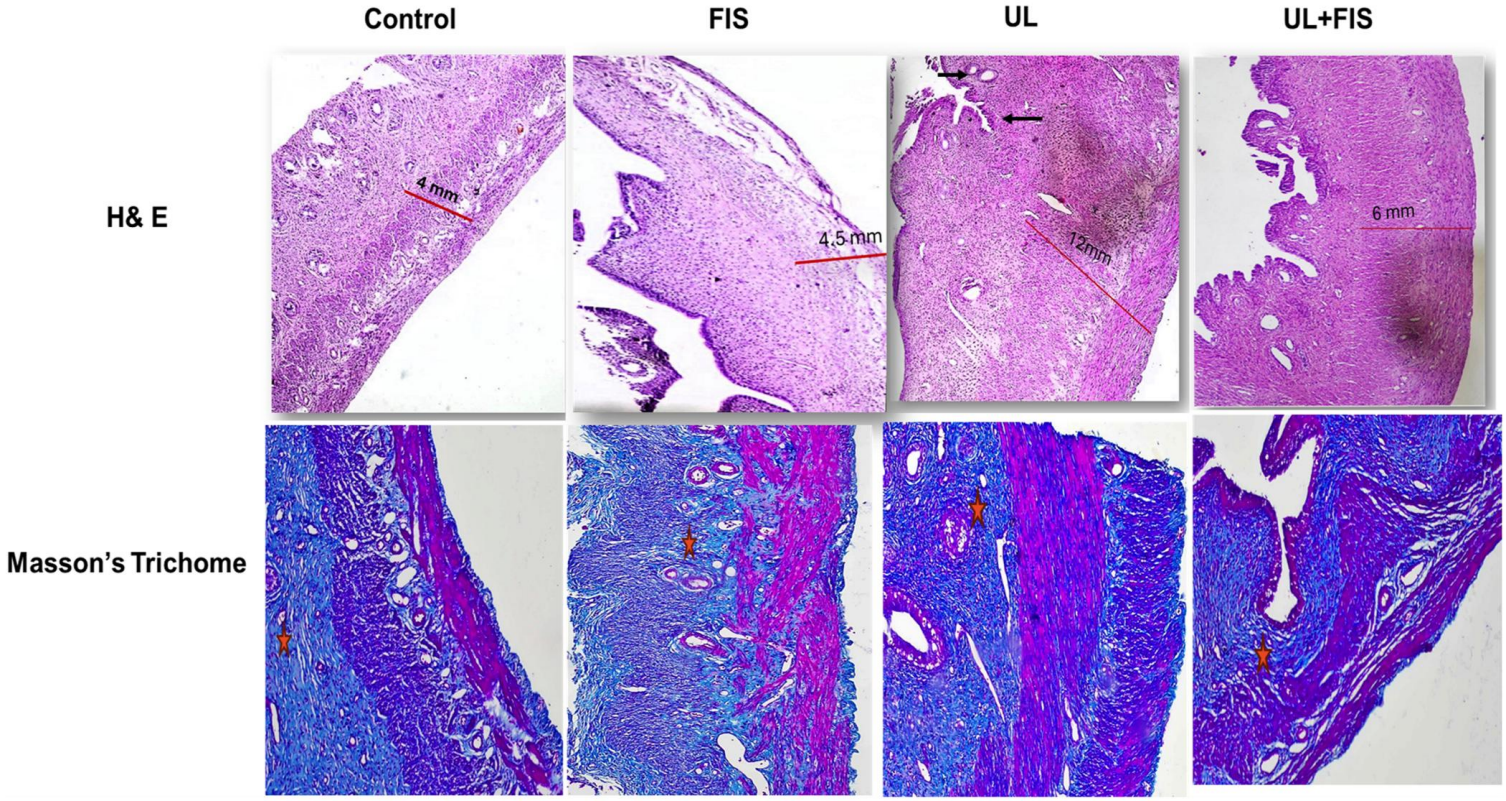
Light microscopic examination of H&E and Masson's trichrome‐stained sections of the uterine tissues from different groups (x40). Control group showing normal intact uterine layers (Endometrium, myometrium, and perimetrium) without hyperplasia, hypertrophy, or any inflammatory reaction. myometrial thickness is about 4 mm (redline). The FIS group shows myometrial thickness of about 4.5 mm (red line). Rats of the induction group show an increase in endometrial thickness and hyperplasia (arrow) as well as eosinophilic infiltration within the epithelial cells. The myometrial smooth muscle cell also shows increased thickness up to 12 mm, accompanied by disarrangement and pinkish homogenous material (hyaline degeneration (redline) with mononuclear inflammatory cells infiltrating muscle fibers. The treated group shows myometrial thickness of about 6 mm (red line). Masson's trichrome staining demonstrates mild collagen fiber deposition in the control & FIS groups, respectively (astres). In the induction group, marked collagen fiber deposition is observed around the uterine gland blood vessels (astres). The UL + FIS group shows moderate collagen deposition around the uterine glands & the blood vessels (astres).

Sections of the uterine tissues of different animal groups were stained with H&E and Masson trichrome (MT) stains. Regarding H& E‐stained sections, the uterine tissues of the control group showed normal intact uterine layers (Endometrium, myometrium, and perimetrium) without hyperplasia, hypertrophy, or any inflammatory reaction, with myometrial thickness of about 4 mm. on the other hand, the uterine tissues of the model group showed increase in endometrial thickness, endometrial hyperplasia, vacuolar degeneration, and eosinophilic infiltration within the epithelial cells. Myometrial smooth muscle cells also showed increased thickness up to 12 mm, accompanied by disarrangement and pinkish homogenous material (hyaline degeneration), as well as mononuclear inflammatory cells infiltrated into muscle fibers. Regarding the treated group, the uterine tissues had less hyperplastic with little hyaline degenerated myometrial smooth muscle cells, little inflammation, and showed a marked decrease of myometrial thickness to about 6 mm (Figure 6).

## 4 Discussion

Uterine leiomyomas (UL) represent the most frequently occurring benign tumors in the uterus and are one of the leading indications for hysterectomy worldwide in reproductive‐aged women [1]. Their development and progression are driven by excessive myometrial cell proliferation, chronic inflammation, and increased extracellular matrix (ECM) accumulation [4]. Although UL has a major clinical impact, there are no reliable treatments that preserve both the uterus and fertility. Current treatments are often costly, can cause substantial side effects, and have high recurrence rates. Therefore, there is an urgent need for safer, more affordable options that can shrink fibroids while preserving the uterus [2].

Current pharmacological therapies of ULs, including gonadotropin‐releasing hormone (GnRH) agonists and antagonists, selective progesterone receptor modulators (SPRMs), combined oral contraceptives (COCs), androgenic steroids and aromatase inhibitors, are mainly intended for short‐term use. However, their long‐term use is frequently limited by incomplete efficacy and treatment‐related adverse effects [27, 28]. GnRH agonists are associated with hypoestrogenic symptoms and bone mineral loss, whereas GnRH antagonists, despite avoiding the initial flare effect, remain linked to adverse events such as abnormal uterine bleeding, headache, nausea, decreased libido, and reduced bone mineral density [29]. Likewise, androgenic agents and aromatase inhibitors are associated with androgenic and metabolic adverse effects, while SPRMs have raised major safety concerns, including severe hepatotoxicity, leading to restriction of their clinical use [28, 30]. Furthermore, COCs mainly alleviate abnormal uterine bleeding without significantly reducing fibroid size [31]. Collectively, these limitations underscore the need for safer therapeutic alternatives capable of targeting the underlying pathogenic mechanisms of ULs.

Naturally occurring dietary compounds, in either purified or chemically modified forms, are gaining attention as potential therapeutic agents in cancer research [32]. Among these novel dietary agents, fisetin, a flavonoid widely distributed in fruits and vegetables, has shown strong anticancer promise because of its wide range of biological activities [33]. A previous in vitro study reported that fisetin induces apoptosis and suppresses the proliferation of UL cells through multiple molecular pathways [34]. The present study extends these findings by evaluating the therapeutic potential of fisetin, a compound known for its anti‐proliferative, anti‐inflammatory, and antifibrotic effects, in a rat model of UL induced by Monosodium glutamate (MSG). MSG, a commonly used flavor enhancer, has been reported to reproduce key hormonal and histopathological features of the UL in experimental models [18].

Although the present study did not include a dedicated toxicological assessment, no mortality or overt signs of toxicity were observed in fisetin‐treated rats during the treatment period. The selected oral dose (50 mg/kg/day) was based on previous studies demonstrating its efficacy and favorable tolerability in rodents [23, 35, 36]. Consistent with our findings, repeated oral administration of fisetin at this dose has been reported to produce no treatment‐related adverse effects [35], while acute and chronic toxicity studies have demonstrated a wide safety margin [37, 38]. Moreover, the reported acute oral LD_50_ of fisetin in rats exceeding 5000 mg/kg body weight indicates that the dose used in the present study is well below toxic levels [39]. Nevertheless, additional comprehensive toxicological studies, including long‐term safety evaluations, are warranted to further characterize the safety profile of fisetin before its potential clinical application.

The results of the current study demonstrated that MSG administration significantly elevated serum levels of estradiol and progesterone, especially at the termination of the induction phase. The data align with the widely accepted concept that leiomyomas are hormone‐dependent tumors driven by the combined actions of estrogen and progesterone [2, 24]. Similarly, prior investigations indicate that MSG increases estrogen and progesterone, contributing to conditions that promote UL development [40, 41]. MSG elevates estrogen and progesterone levels by stimulating aromatase activity, which converts androstenedione into estradiol, and by increasing cholesterol, the precursor of steroid hormones, thereby enhancing UL cell proliferation [41, 42].

The induction of UL was evident from the marked increases in uterine weight, uterine coefficient, and myometrial thickness, which are indicators of UL growth [43]. These structural changes occurred alongside the elevated hormonal levels, confirming successful induction of UL. According to our findings, the uteri of model animals exhibited increased myometrial thickness, reaching up to 12 mm, indicating myometrial hypertrophy and hyperplasia. Additionally, Masson's trichrome staining exhibited excessive collagen deposition, confirming the development of fibrosis in the model group.

The observed increase in uterine weight in rats likely results from estrogen and progesterone‐driven myometrial hyperplasia and hypertrophy. Estrogen, the primary mitogenic factor of the uterus, stimulates myometrial cell proliferation and enhances the expression of progesterone receptors, thereby increasing the tissue's responsiveness to progesterone [41]. Progesterone further promotes UL growth by enhancing both cell proliferation and tumor size via the upregulation of proliferating cell nuclear antigen (PCNA), and it also drives fibrotic remodeling by promoting ECM deposition, including collagen [44].

Notably, treatment with fisetin markedly reduced serum concentrations of estradiol and progesterone, accompanied by a significant decrease in uterine size, myometrial thickness, and collagen content. These effects may be explained, at least in part, by the downregulation of CYP17A1, a key enzyme involved in steroidogenesis, as reported in a previous study, in which fisetin significantly reduced both CYP17A1 mRNA and protein expression in ovarian tissue of a PCOS rat model [45]. However, as CYP17A1 expression was not evaluated in the present study, this proposed mechanism remains speculative. The concurrent decreases in myometrial thickness and ECM deposition may reflect the anti‐fibrotic effects of fisetin and may also suggest reduced myometrial cell proliferation.

Transforming growth factor β (TGF‐β) is a multifunctional polypeptide composed of three major isoforms: TGF‐β1, TGF‐β2, and TGF‐β3. It plays a critical role in UL development and growth. Activation of multiple pathways in UL enhances TGF‐β production and release, accelerating ECM accumulation and supporting tumor growth, which collectively underlie several clinical manifestations [46]. Overexpression of TGF‐β1 can trigger the steroid hormone‐dependent PI3K/AKT signaling pathway in fibroblasts, driving their transition into α‐SMA‐expressing myofibroblasts. This transformation further amplifies ECM deposition and drives more extensive fibrotic remodeling in the tumor microenvironment [47].

The model group in this study showed significantly higher expression of TGF‐β1 and α‐SMA in uterine tissues compared to the control group. Masson's trichrome staining further demonstrated irregular collagen deposition around uterine glands and blood vessels, supporting the presence of fibrosis. These findings are consistent with recent clinical and experimental studies showing that the higher TGF‐β1 and α‐SMA expression is linked to enhanced fibrotic remodeling in UL specimens [48, 49]. This suggests that MSG may promote structural changes resembling leiomyoma pathology, partly through activation of TGF‐β1 and increased α‐SMA expression, reflecting enhanced myofibroblast activation and ECM accumulation

On the other hand, administration of fisetin markedly decreased TGF‐β1 and α‐SMA expression in uterine tissues of the UL + FIS group. Masson's trichrome staining further supported these molecular findings by showing decreased collagen deposition around uterine glands and blood vessels. These findings agree with earlier studies showing that fisetin can effectively decrease TGF‐β1 and α‐SMA expression in experimental models of endometriosis and hepatic fibrosis, further supporting its anti‐fibrotic potential [21, 50].

HMGB1 is a highly conserved nuclear protein located in the nucleus. It is essential for eukaryotic cell function, contributing to processes such as DNA recombination, repair, and replication [9]. On the other hand, HMGB1 is critically involved in promoting inflammation and fibrotic processes, both of which are key contributors to the pathogenesis of UL. When HMGB1 binds to toll‐like receptors 2 and 4, it triggers the NF‐κB signaling pathway. This increases the production of inflammatory cytokines and amplifies fibrogenic responses [51]. Moreover, HMGB1 is overexpressed in various carcinomas, where it promotes angiogenesis and tumor cell proliferation, highlighting its pro‐oncogenic, antiapoptotic role [9, 11].

Notably, the HMGB1‐TGF‐β1 axis represents a major driver of fibrosis, forming a self‐reinforcing loop. HMGB1 promotes myofibroblast differentiation and collagen synthesis by upregulating TGF‐β1 expression and activating its downstream signaling pathways, enhancing α‐SMA expression and fibrotic remodeling. On the other hand, TGF‐β1 can reciprocally induce HMGB1, further sustaining inflammation and fibrogenesis [52].

The present findings demonstrate a significant upregulation of HMGB1 and pro‐inflammatory cytokines (IL‐1β and TNF‐α) in the uterine tissues of the induction group compared with normal controls. In agreement, previous studies have shown upregulated expression of HMGB1 and proinflammatory cytokines in UL pathology [12, 53]. On the other hand, fisetin treatment significantly downregulated the expression of HMGB1, IL‐1β, and TNF‐α in the animals with UL. These results are consistent with previous studies, which further support the anti‐inflammatory potential of fisetin through inhibition of HMGB1 and its downstream inflammatory mediators [21, 54]. Collectively, these results suggest fisetin may help modulate HMGB1‐driven inflammatory pathways involved in UL development.

The S1P/SphK1 pathway regulates various biological processes and is upregulated in cancer, promoting tumor progression [16]. S1p, a bioactive sphingolipid metabolite, also drives fibrotic and inflammatory responses, which contribute to gynecological disorders such as endometriosis, adenomyosis, and UL [15]. Interestingly, sphingolipids can amplify estrogen‐driven signaling, enhancing the uncontrolled growth of UL. Estrogen activates SphK1, increasing the intracellular concentration of S1P and supporting the proliferation and survival of cancer cells. S1P was also found to be released by tumor cells employing ABC‐transporters ABCC1 (ATP‐binding cassette subfamily C member (1) and ABCG2 (ATP‐binding cassette subfamily G member (2) after stimulation by estradiol [55].

There is evidence of bidirectional cross‐talk between the TGF‐β1 and S1P signaling pathways. The Sphk1‐S1P‐SIRPs axis and TGF signaling interact in a profibrotic feedback loop. Sphk1 converts sphingosine to S1P, which is exported by ABC proteins. Extracellular S1P binds to SIRP2 and SIRP3, activating Smad signaling, while TGF‐β1/Smad signaling enhances Sphk1 expression, further reinforcing the axis. The S1P gradient also recruits immune cells, like macrophages and lymphocytes, increasing TGF‐β1 secretion. Together, these interactions amplify fibrogenesis, and targeting this axis may represent a potential therapeutic strategy for fibrotic disorders like UL [56].

In the current study, the UL‐induced rats exhibited noticeably higher serum S1P and greater SphK1 expression in the uterine tissues than healthy controls. This supports previous findings indicating that S1P signaling dysregulation, mainly through SphK1 and SphK2, contributes to UL pathogenesis by promoting ECM deposition, fibroblast‐to‐myofibroblast differentiation, and activin‐driven fibrosis [57]. Interestingly, fisetin significantly lowered serum S1P levels and uterine SphK1 expression, likely by reducing estradiol levels and TGF‐β1 expression, which in turn may inhibit SphK1 activation. In line with these findings, Zhou et al. showed that fisetin suppresses SphK1 signaling, mitigates fibrotic changes, and alleviates cellular stress, indicating its promise as an anti‐fibrotic and cytoprotective compound [58].

Notably, a functional interaction between SphK1 and HMGB1 has also been demonstrated. Inhibiting SphK1 suppresses HMGB1 expression and its nuclear‐cytoplasmic translocation, preventing its extracellular release and the subsequent activation of inflammatory responses [17]. Collectively, the coordinated pathways, TGF‐β‐ HMGB1‐S1P, have been suggested to play a central role in the development of UL by driving overgrowth, inflammation, and fibrotic changes, pointing to promising therapeutic strategies.

Despite the promising findings, certain limitations should be acknowledged. Although fisetin treatment was associated with modulation of S1P/SphK1, TGF‐β, and HMGB1‐mediated inflammatory pathways, mechanistic investigations using targeted functional approaches, such as receptor antagonists, gene silencing, or gene overexpression, are needed to confirm the direct involvement of these pathways in mediating fisetin's therapeutic effects. In addition, because comparative efficacy was beyond the scope of the present study, no direct comparisons with established pharmacological treatments were included. Subsequent preclinical investigations with clinically relevant standard therapies as positive controls would enable a more comprehensive evaluation of the relative efficacy and translational potential of fisetin. Furthermore, because all animals were euthanized immediately after competition of the treatment period, the durability of fisetin's therapeutic effects after treatment cessation could not be evaluated. Further studies incorporating an extended post‐treatment follow‐up are warranted to determine whether these beneficial effects are sustained over time. Finally, validation in clinically relevant models, followed by well‐designed clinical trials, is required to establish the therapeutic potential and long‐term safety of fisetin before its clinical translation.

In conclusion, our findings indicate that fisetin ameliorated hormonal imbalance, inflammation, fibrosis, and uterine enlargement in the MSG‐induced rat model of UL. These beneficial effects were associated with modulation of key signaling pathways involved in leiomyoma pathophysiology, including S1P/SphK1, TGF‐β, and HMGB1‐mediated inflammatory pathways, suggesting that this pathway may contribute to the protective effects of fisetin. In the present experimental model, fisetin exhibited favorable anti‐inflammatory, antiproliferative, and antifibrotic properties. Collectively, these findings provide preclinical evidence supporting fisetin as a promising natural candidate for further investigation as a potential therapeutic strategy for UL.

## Author Contributions

**Nashwa Maghraby:** conceptualization, writing – original draft, software, data curation, investigation, formal analysis. **Esraa Khalaf:** methodology, funding acquisition, data curation, investigation, formal analysis, visualization. **Marwa T. Hussien:** methodology, investigation, formal analysis. **Ahmed M.A. Sobh:** data curation, supervision, writing, reviewing, and editing. **Nagla T. El‐Meleg:** conceptualization, supervision, writing, reviewing, and editing.

## Ethics Statement

All study procedures were approved by the Medical Ethics Committee, Faculty of Medicine, Assiut University (approval no. 04‐2023/200221). The manuscript is approved by the authors for publication.

## Conflicts of Interest

The authors declare no conflicts of interests.

## Data Availability

The data that support the findings of this study are available from the corresponding author upon reasonable request.
